# Increased absorbed liver dose in Selective Internal Radiation Therapy (SIRT) correlates with increased sphere-cluster frequency and absorbed dose inhomogeneity

**DOI:** 10.1186/s40658-015-0113-4

**Published:** 2015-04-25

**Authors:** Jonas Högberg, Magnus Rizell, Ragnar Hultborn, Johanna Svensson, Olof Henrikson, Johan Mölne, Peter Gjertsson, Peter Bernhardt

**Affiliations:** Department of Radiation Physics, The Sahlgrenska Academy, University of Gothenburg, SE-41346 Gothenburg, Sweden; Department of Surgery, Sahlgrenska University Hospital, SE-41346 Gothenburg, Sweden; Department of Oncology, Sahlgrenska University Hospital, SE-41346 Gothenburg, Sweden; Department of Radiology, Sahlgrenska University Hospital, SE-41346 Gothenburg, Sweden; Department of Pathology, Sahlgrenska University Hospital, SE-41346 Gothenburg, Sweden; Department of Clinical Physiology, Sahlgrenska University Hospital, SE-41346 Gothenburg, Sweden; Department of Medical Physics & Biomedical Engineering, Sahlgrenska University Hospital, SE-41346 Gothenburg, Sweden

**Keywords:** Radioembolisation, Y-90, SIRT, Liver, Dosimetry, Microspheres

## Abstract

**Background:**

The higher tolerated mean absorbed dose for selective internal radiation therapy (SIRT) with intra-arterially infused ^90^Y microspheres compared to external beam therapy is speculated to be caused by absorbed dose inhomogeneity, which allows for liver regeneration. However, the complex liver microanatomy and rheology makes modelling less valuable if the tolerance doses are not based on the actual microsphere distribution. The present study demonstrates the sphere distribution and small-scale absorbed dose inhomogeneity and its correlation with the mean absorbed dose in liver tissue resected after SIRT.

**Methods:**

A patient with marginally resectable cholangiocarcinoma underwent SIRT 9 days prior to resection including adjacent normal liver tissue. The resected specimen was formalin-fixed and sliced into 1 to 2-mm sections. Forty-one normal liver biopsies 6-8 mm in diameter were punched from these sections and the radioactivity measured. Sixteen biopsies were further processed for detailed analyses by consecutive serial sectioning of 15 30-μm sections per biopsy, mounted and stained with haematoxylin-eosin. All sections were scrutinised for isolated or conglomerate spheres. Small-scale dose distributions were obtained by applying a ^90^Y-dose point kernel to the microsphere distributions.

**Results:**

A total of 3888 spheres were found in the 240 sections. Clusters were frequently found as strings in the arterioles and as conglomerates in small arteries, with the largest cluster comprising 453 spheres. An increased mean absorbed dose in the punch biopsies correlated with large clusters and a greater coefficient of variation. In simulations the absorbed dose was 5–1240 Gy; 90% were 10-97 Gy and 45% were <30 Gy, the assumed tolerance in external beam therapy.

**Conclusions:**

Sphere clusters were located in both arterioles and small arteries and increased in size with increasing sphere concentration, resulting in increased absorbed dose inhomogeneity, which contradicts earlier modelling studies.

## Background

The use of selective internal radiation therapy (SIRT) with intra-arterially infused ^90^Y spheres to treat liver metastases and primary liver malignancies has prompted a need for further understanding of normal liver parenchyma tolerance, which is not transferable from external beam therapy [1]. The higher mean tolerated absorbed dose is thought to be due to a lower dose rate and an inhomogeneous small scale dose distribution, allowing for regeneration [1]. However, the complex liver microanatomy and rheology of infused microspheres makes modelling of microsphere distribution, absorbed dose distribution, and tolerance doses less valuable if it is not based on a reasonably accurate microsphere distribution.

As Cremonesi et al. described in their recent review [1], valid dosimetric calculations for SIRT is a complex issue given our limited knowledge of microsphere distributions on a microscopic and, in clinical contexts, without dosimetry based on post-therapeutic imaging [2], also on a macroscopic level. When applying dosimetric calculations to radioembolisation treatment (RE) with resin (SIR-Spheres®, SIRTex Medical Limited Sydney, Australia) or glass (Therasphere® BTG, Ontario, Canada) spheres, uniform activity distributions are routinely assumed, which is practical when reporting liver tolerance for large patient groups [3-19]. As RE treatments and follow-up studies have increased during the last two decades, some characteristics have become obvious, such as the tolerance of a higher mean absorbed dose to normal liver parenchyma and a higher threshold for radiation-induced liver disease (RILD) compared to external beam radiation therapy (EBRT), which cannot be explained solely by the lower dose rate [1,9,20,21].

The non-uniform absorbed dose distribution resulting from a heterogeneous activity distribution is most certainly an important factor [1,5,22-33]. Therefore, several studies have opted to investigate and simulate the situation in a more realistic way with heterogeneous distributions on a macroscopic or microscopic level. On the macroscopic level, post-therapeutic ^90^Y bremsstrahlung imaging has the poorest resolution and contrast recovery [22,33-35], followed by pre-therapeutic ^99m^Tc-macro-aggregated albumin (^99m^Tc-MAA) SPECT [23-25] and PET imaging, which is possible due to the small, but sufficient, contribution of internal pair production for positron emission [5,26,27,36-42]. However, the resolution of these systems is beyond the limit of detecting microsphere non-uniformity and investigations determining the influence of heterogeneous small-scale dosimetry on liver tolerance.

On the small scale, single microspheres and clusters have been studied by microscopy of explanted tissue [24,25,28-31]. However, these studies included a limited number of samples and focused mainly on microsphere distributions around and within tumour tissue. Therefore, the novel small scale liver dosimetry modelling performed by Gulec et al. [43] and Walrand et al. [26,27] was forced to use assumed microsphere distributions. Gulec et al. simulated absorbed dose distributions based on an assumed microanatomy surrounding the microspheres, which were proposed to be uniformly located in the artery in the portal tract. Walrand et al. extended this rigid liver model to a simulation model of non-uniform microsphere distributions and absorbed doses. Non-uniform distribution of microspheres in the final portal arterioles was achieved by simulating the microsphere path through a model of the liver artery tree with 21 branching nodes using symmetric or asymmetric branching probabilities for the microspheres. These simulations resulted in a broad absorbed dose distribution, which was broader for glass compared to resin microspheres due to the use of fewer glass microspheres. This expected theoretical phenomenon for random spreading through the arterial tree was also pointed out by Chiesa et al. [44] and Spreafico et al. [45], who also addressed that the biological effect will increase with decreasing specific activity due to the increased number of microspheres, resulting in a more uniform absorbed dose. However, these authors also addressed that this would be theoretically correct if no stasis occurs; i.e., no trapping in the larger arterioles or small arteries located upfront in the arterial tree.

In a recent study [23], we reported the activity distribution of marginally resected, radioactive, cholangiocarcinoma tumour tissue and the surrounding normal liver parenchyma [22] using autoradiography, biopsy activity measurements, and microscopy of sectioned biopsies. The activity concentrations within the liver parenchyma were heterogeneous on a scale larger than the mean range of the beta electrons, and clusters of different sizes were found in portal arterioles, as well as larger arterioles and small arteries of the liver parenchyma. In the studied biopsies, the majority of spheres were trapped in small arteries with cluster sizes of up to 59 spheres per cluster. Such trapping will generate a complicated microsphere distribution in which it is not obvious if the width of the microsphere and absorbed dose distribution will decrease or increase with the number of injected microspheres.

Because upfront clustering in the arterial tree may generate a systematic difference in the distribution of the absorbed dose throughout the parenchyma, with more dramatic dose gradients than previously expected [24-31], we aimed to describe the microsphere distribution in small arterioles and the clustering in larger arterioles or small arteries. In the present study, we aimed to investigate a larger sample of biopsies microscopically, spanning a broader spectrum of mean activity concentrations, as compared to our previous study [23]. The rationale was to study the frequency of microsphere clusters and the distribution of microsphere cluster sizes in relation to the mean activity concentrations within individual biopsies. Furthermore, we aimed to simulate the small-scale absorbed dose distribution and investigate to what extent found non-uniformities would be homogenised by cross-dose (cross-fire) effects [46].

## Methods

### Patient and clinicopathology

A female aged 62 years who suffered from a marginally resectable cholangiocarcinoma accepted neo-adjuvant treatment with ^90^Y-labelled microspheres (i.e., SIRT) followed by liver surgery [22,23].

Pre-study investigations revealed tumour masses of 37 g and 56 g in the left and right lobes, respectively, with corresponding normal liver parenchyma of 700 g and 1350 g, respectively. According to standard procedures, the patient was first examined by selective hepatic artery angiography and artery coiling, followed by ^99m^Tc-MAA using planar imaging and SPECT/CT with a GE Millenium VG camera (energy window 126-154 keV) and high resolution collimator to evaluate the hepatic distribution and degree of pulmonary shunting.

Two weeks later, the hepatic artery was recannulated for infusion of the ^90^Y-labelled SIR-Spheres (Sirtex Medical Limited, North Sydney, Australia). The microspheres consist of polymeric resin with an approximate activity of 50 Bq (40–70 Bq) per sphere and mean diameter of 30 μm (20–60 μm). The mean and maximum ranges of the electrons emitted from ^90^Y (mean energy, 0.934 MeV) are 2.5 mm and 11 mm, respectively, in human soft tissues, and the physical half-life is 64 h [14,47-49]. The number of spheres suspended in 30–40 ml of distilled water and injected in less than an hour was approximately 30 million (1.6 GBq). The distribution of ^90^Y microspheres was recorded by bremsstrahlung detection applying an energy window of 55–285 keV using a medium energy (ME) collimator [22]. Nine days later the patient underwent liver surgery with an ultrasonic cavitron aspirator, including resection of the tumour masses and a rim of surrounding normal liver tissue.

### Analytical procedures

The resected specimens were immersed in isotonic formaldehyde (10%) for 48 hours and machine sliced into 1 to 2-mm-thick sections. Some sections were subjected to autoradiography; other sections were punched into 41, 6 to 8-mm-diameter, biopsies, weighed, and the activity measured (^90^Y bremsstrahlung in a gamma well counter, Wizard® 1480, PerkinElmer, Waltham, MA, USA) in vials with 1 ml formaldehyde solution [23].

After measuring the activity, 16 of the 41 biopsies were paraffin embedded and 15 consecutive 30-μm thick circular sections were obtained from each of the 16 punch biopsies of normal liver tissue (240 sections total). These sections were haematoxylin-eosin stained. Each section was microscopically investigated for microspheres, which were counted and classified as single or clustered. A cluster was defined as 2 or more spheres, with an inter-sphere distance of 200 μm (i.e. 7 sphere diameters) or less [23] irrespective of strings in smaller vessels or conglomerates in larger vessels, which sometimes extended throughout the 15 sections. The number of spheres in each cluster was also counted.

### Dosimetry issues

The absolute number of spheres within the sample created by the 15 30-μm sections (representing approximately ¼ - ½ of each punch biopsy) was counted and categorised regarding number of connected spheres (cluster size). The distributions of microspheres found microscopically, for every of the individual 16 sectioned volumes, were used for simulation of small-scale dose distributions. First, each activity locus (single sphere or cluster) was selected on random, from the biopsy-unique microsphere distribution, and then pre-positioned on random in a 160 × 160 × 1 matrix, with spatial resolution 452 μm (resolution adapted to dose point kernel, as described in the next paragraph). The randomised selection and pre-positioning of activity loci was repeated until the volume had reached the same (well chamber activity-measured) mean sphere concentration as for the entire biopsy, from which the sectioned sub-volume had been collected. By this process, deviation from the biopsy mean activity concentration, within the sectioned sub-sample, had no influence over the final simulated sphere concentration. This randomisation process was repeated independently, 160 times, resulting in a final 160 × 160 × 160 voxel volume, where every layer was uniquely randomised, resulting in 160 layers with almost the same sphere concentration, similar frequency, regarding activity locus size, but with a high probability of uniqueness in spatial distribution of the activity loci. The entire process was repeated, for all biopsy subsamples, resulting in 16 different 160 × 160 × 160 voxel volumes, totally unique, as the volumes were both randomised, from biopsy-specific cluster patterns (distributions), found microscopically in the sectioned sub-volumes, as well as adjusted to unique microsphere concentrations, based on previous well camber activity measurements, for the 16 biopsies.

The simulated 16 volumes were convolved individually with a ^90^Y dose point kernel containing 53 × 53 × 53 voxels, with a spatial resolution of 452 μm, according to Prestwich et al. [50] (the same resolution for volume and kernel), tuning the absorbed fraction of the centremost voxels according to Siegel and Stabin [51]. Absorbed dose distributions, in which absorbed dose equilibrium was obtained, were used as output, i.e., the centremost 150,000 voxels, corresponding to a 24 × 24 × 24 mm^3^ cube or 14 cm^3^ of tissue, for each of the 16 biopsies.

### Statistical analysis

Median, mean ± standard deviation and the coefficient of variation (CV) were used to describe normally distributed continuous variables. Possible relationships between variables were evaluated by linear regression. For all statistical analyses, the statistical software IBM SPSS® 19 was used, applying linear regression by Ordinary Least Squares (OLS), to either the dependent variable data point set or (when testing exponential dependence) the corresponding logarithmised data point set. Hypothesis tests (two-sided *t*-tests) were performed and *p* ≤ 0.05 was considered significant.

## Results

**Figure 1:**
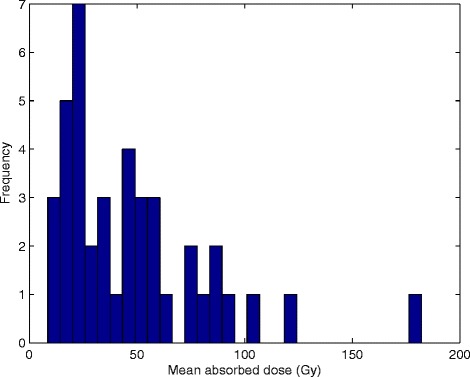
**Absorbed dose distribution observed in normal liver parenchyma.** The mean activity in the 41 punch biopsies (29 to 88 mg) at the time of treatment was 950 Bq/mg, resulting in a mean absorbed dose of 47 ± 34 Gy; CV 0.73 and a median absorbed dose of 38 Gy assuming local energy deposition (range 8-182 Gy). The 10^th^, 25^th^, 75^th^, and 90^th^ percentiles were found at 17, 24, 59, and 88 Gy.

Planar and SPECT/CT imaging of the ^99m^Tc-MAA infusions revealed a pulmonary shunting of 3.5% and tumour-to-normal liver activity concentration (TNC) of 3.8. The expected mean dose to normal liver was 33 Gy for the planned SIRT activity. The activity histogram and the distribution parameters of the 41 punch biopsies is shown in Figure 1. In the subsequently sectioned and paraffin embedded 16 punch biopsies, the gamma well counter measurements showed a mean absorbed dose of 8, 12, 17, 19, 21, 24, 29, 33, 38, 46, 48, 54, 56, 59, 61, and 84 Gy.

**Figure 2:**
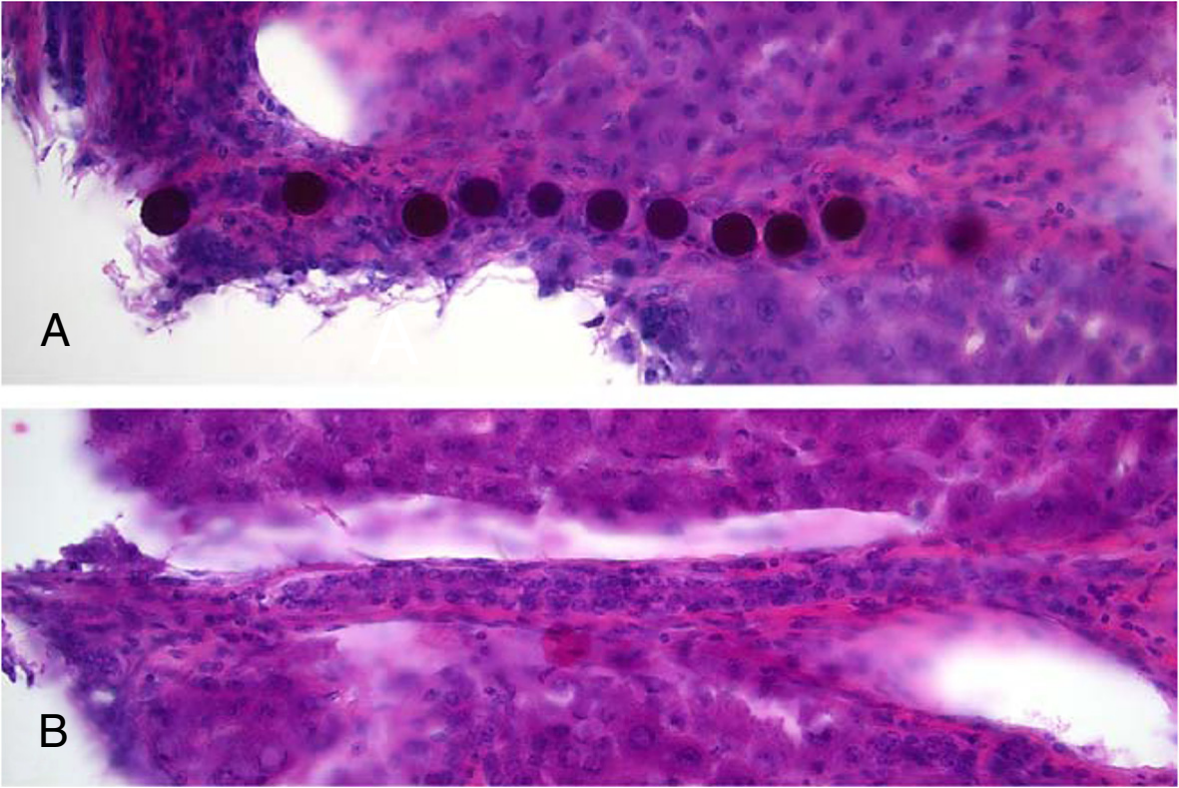
**Light microscopy of filled and empty arterioles.** Single spheres and clusters with a string pattern were observed in the arterioles. The images show a string of microspheres in an arteriole **(A)** and an empty arteriole of the same size **(B)**.

**Figure 3:**
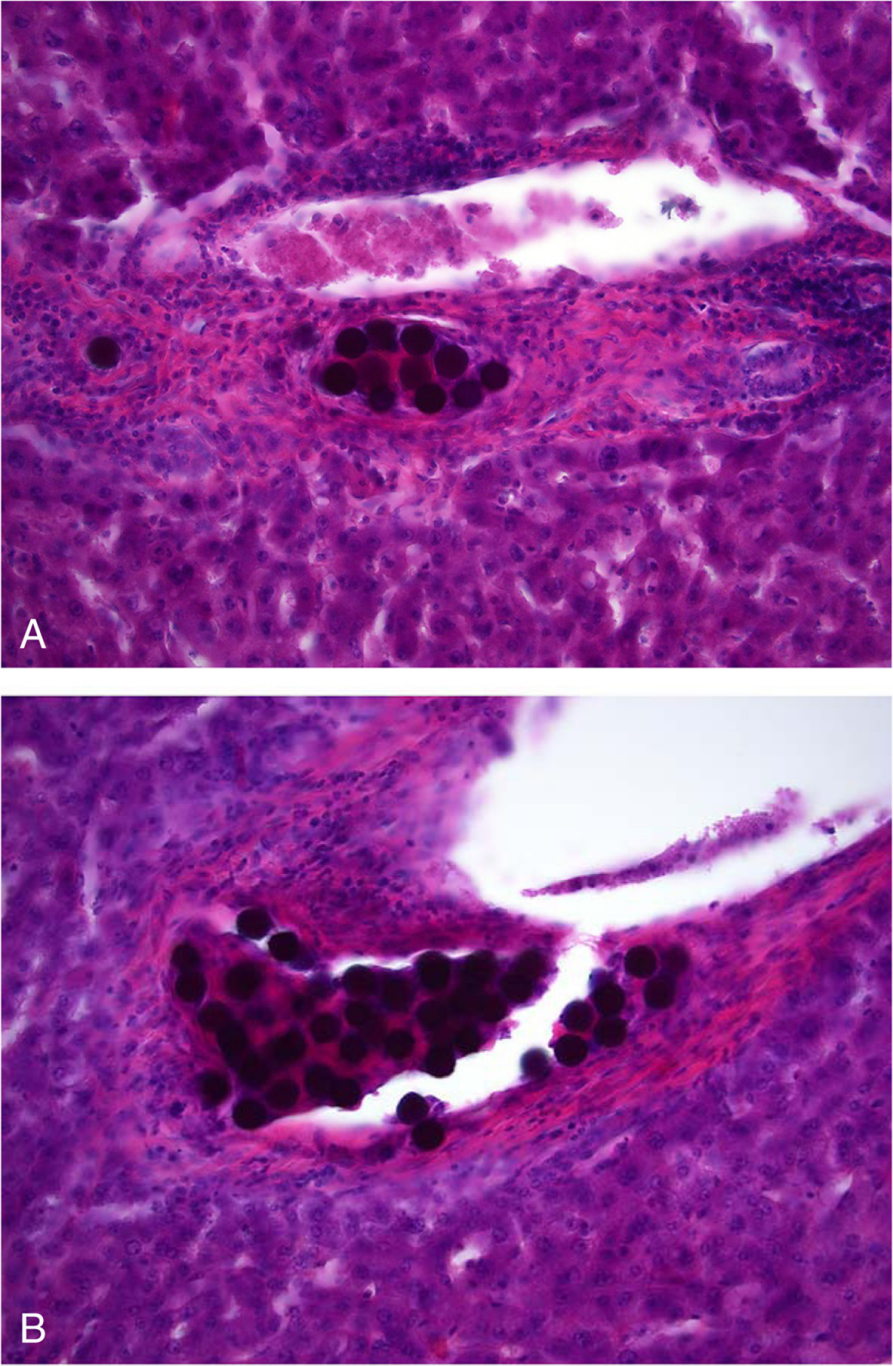
**Light microscopy of sphere clusters in different-sized arteries.** The images show **(A)** a mid-sized sphere cluster (13 of the 35 spheres in this section) within a small artery and **(B)** a larger artery with a massive cluster (44 of the 306 spheres in this section). Larger bulky clusters were gathered in small arteries. The three largest clusters (174, 306, and 453 spheres per cluster) were found in smaller arteries within the three biopsies with the highest mean absorbed dose, i.e., 59, 61, and 84 Gy.

**Figure 4:**
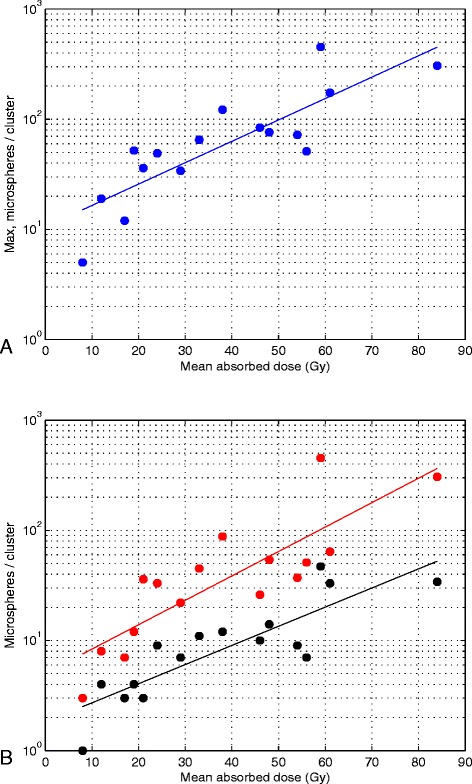
**Exponentially increasing cluster size with increasing mean absorbed dose.** **(A)** The largest cluster size in each of the 16 biopsies and the corresponding trendline for exponential increase (R^2^ = 0.70, *p* < 0.05, two-sided *t* test), from approximately 16 spheres per cluster at 10 Gy to 400 spheres per cluster at 80 Gy. **(B)** The spheres are divided into three cluster size intevals in which the spheres in the biopsies were gathered. Twenty-five percent of the microspheres for a specific mean absorbed dose were found within clusters of the same size or smaller than the level indicated by a black dot, 25% of the microspheres were found within the cluster size interval between the absorbed-dose-specific black dot and the corresponding absorbed-dose-specific red dot, and the remaining 50% of the microspheres were found within clusters of the same size or larger than the size indicated by the absorbed-dose-specific red dot. The cluster size levels increase exponentially with the mean absorbed dose in the biopsies (black dot level: R^2^ = 0.72, *p* < 0.05, two-sided *t* test; red dot level: R^2^ = 0.70, *p* < 0.05, two-sided *t* test).

**Figure 5:**
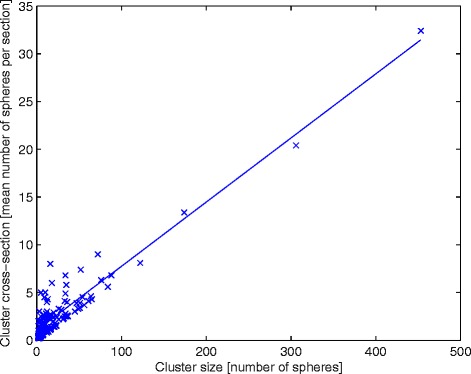
**Linear relationship between cluster size and cluster cross-section.** 277 clusters found within the 16 biopsies. There is a linear increase in cluster cross-section size (mean number of spheres per section) with cluster size (number of spheres per cluster) (R^2^ = 0.87, *p* < 0.05, two-sided *t* test).

Different microsphere clustering patterns were seen with light microscopy (Figures 2 and 3). The majority of the clusters were distributed through more than three sequential sections, and several strings of microspheres were distributed through the 15 sections, indicating that some clusters may be even larger than discovered. Figure 4 shows the tendency of exponentially increasing cluster size with mean absorbed dose per biopsy. Figure 5 shows a linear increase in cluster cross-section size with cluster size.

**Figure 6:**
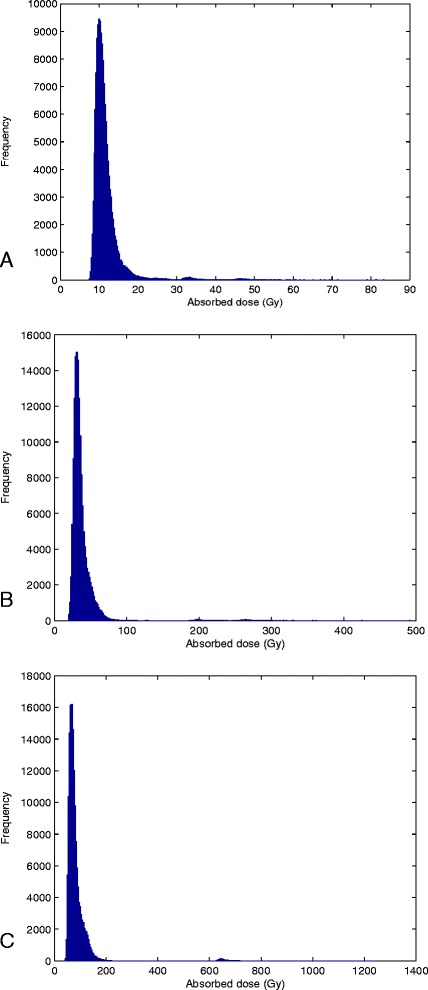
**Histogram of the simulated voxel absorbed dose distributions of three biopsies.** Mean absorbed doses, based on activity measurements, as well as CV from simulations: **a)** 12 Gy; CV 0.35 **b)** 38 Gy; CV 0.53 and **c)** 84 Gy; CV 0.66. The ratios of simulated/measured mean absorbed dose are 0.98, 0.98 and 0.99, respectively.

**Figure 7:**
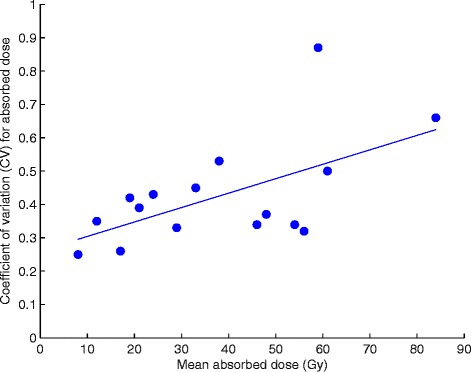
**The CV of absorbed dose increasing with mean absorbed dose.** The 16 separate absorbed dose simulations were based on cluster distribution patterns of the microscopic sub-samples, but added up to the mean absorbed dose within the entire 16 biopsies, the latter based on activity measurements with gamma well chamber. The resulting graph shows a linear increase of CV of absorbed dose, with mean absorbed biopsy dose (R^2^ = 0.34, *p* < 0.05, two-sided *t* test).

**Figure 8:**
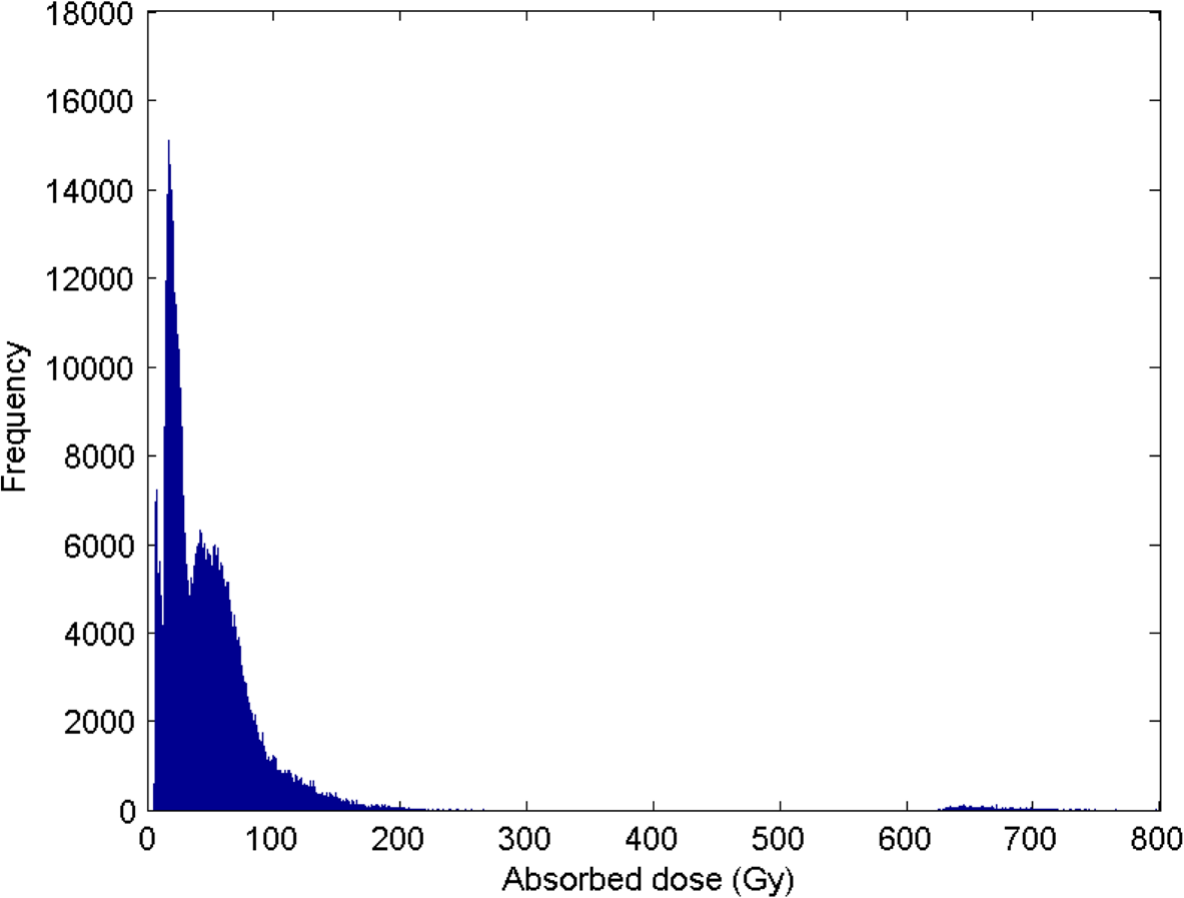
**Histogram of 16 separate simulations, weighted to macroscopic distribution.** The histogram reflects the microscopic absorbed dose distributions (per voxel) in 16 biopsies weighted according to macroscopic mean activity concentration measurements in 41 biopsies, shown in Figure 1. The mean absorbed dose was 43 ± 38 Gy; CV 0.89, the median absorbed dose 36 Gy, and the 10^th^, 25^th^, 75^th^, and 90^th^ percentiles at 15, 20, 58, and 79 Gy. A tail of high absorbed doses are found between 145 and 1200 Gy; this will only affect 1% of the total parenchyma volume, whereas 45% of the parenchyma volume will achieve absorbed doses <30 Gy. 90% of the voxels were found from 10 to 97 Gy. A gathering of voxels was observed in the interval of 600 to 800 Gy, but these voxels formed only 0.17% of the total volume. As the few voxels found above 800 Gy are not visually discernible, the histogram is truncated, accordingly.

Three examples of the simulated absorbed dose distribution in single biopsies are shown in Figure 6. These examples demonstrate an increase in the absolute and relative width of the dose distribution with mean absorbed dose. The adaption ratio of simulated/measured mean absorbed dose was on average 0.99 ± 0.025. The CV of the absorbed dose versus the mean absorbed doses, for the 16 biopsies, is presented in Figure 7, showing a linear relationship. Figure 8 shows the total absorbed dose distribution from the simulation with 16 biopsies, based on microscopic observations of microsphere clusters, weighted in order to reflect the macroscopic distribution of the 41 biopsies, shown in Figure 1.

## Discussion

In a recent study [23], we showed that the activity concentration throughout normal liver parenchyma is heterogeneous on a macroscopic scale, which is in line with the findings of others [1,5,24-32]. The results in the present study add more variation to the previous results, extending the light microscopy analysis of microsphere distributions and small-scale dosimetry. The analysis of 3888 microspheres distributed in 240 sections demonstrates that the increasing non-uniformity with increasing mean absorbed dose per biopsy is caused by the aggregation of large clusters in small arteries.

This conclusion is strengthened by the plot (Figure 5) of linear increase in cluster cross-section size (mean number of spheres per section) with cluster size (number of spheres per cluster) for the 277 found clusters. The cluster cross-section size is expected to be directly correlated to the vessel trans-axial cross-section area, as larger vessels may contain a larger number of spheres per cluster unit length (i.e. section). Figure 5 further shows that the individual data points never deviate much from the trend line, for larger clusters, but, somewhat more for smaller clusters. The small variation for the largest clusters is a reflection of the fact that they tended to be distributed throughout all, or close to all, of the 15 sections investigated, thus limiting the variation upwards (with a potential that some large clusters are in fact even longer, i.e. larger, regarding sphere number). Downwards, there is of course a physical limit, i.e. clusters with certain size, limited in length, will require a minimum vessel size. The larger variation upwards, for smaller and mid-sized clusters, is a reflection of the occurrence of some shorter mid-sized clusters and many short small clusters.

For the smallest clusters, it is also more common with a deflection on the downside of the trend line. The explanation for this is that in the smaller vessels, the clusters tended to be less heavily condensed, with several clusters lacking spheres in one or more sections (explaining the occurrence of clusters with mean number of spheres per cross-section <1). This is probably caused by a combination of vessel tension and the efficient blocking of liquid (blood plasm) by single spheres. Single spheres, flowing with a certain inter-spherical distance will not be allowed to compress to strings of densely packed spheres; when the relaxed vessel diameter is smaller than the sphere diameter, the spheres are both slowing down, by the tension of the vessel walls. The inter-spherical liquid, which cannot longer pass around the spheres, thus retains the stochastically distributed distance between spheres within small vessels, some spheres entering in strings, others sparsely packed.

In contrast, a large cluster, with more than one sphere in cross-section, can probably grow up-streams, when the downstream end of the cluster has started to slow down. The reason for this is the combination of the higher momentum of the spheres, having higher density than the liquid, and the fact that liquid is allowed to pass between the spheres in the cross-sections, allowing for pressure equilibrium.

Even though some liquid may pass through larger clusters, blood flow blockage will still slow them down and halt them; once entrapped, the large cluster will probably not move substantially, at least not as a unit. The entrapment of spheres in arteries will most certainly hamper sphere accumulation downstream in the arterial tree, and it may even completely block deposits in the final arterioles. In this study, we noted single spheres, as well as a string of spheres in the arterioles (Figure 2a), but we also noted a lack of spheres in a large fraction of the arterioles. Blocking spheres upstream in the arterial tree will cause systematic structural non-uniformity in the sphere distribution and absorbed dose distribution; as our results indicate, it will be stronger in parenchyma volumes with a higher mean absorbed dose, i.e., with a high probability of strong absorbed dose gradients caused by large clusters of microspheres. Gulec et al. [43] reported that, when no cross-fire contributions are considered, liver parenchyma 1 mm from a microsphere receives <1% of the absorbed dose compared to the portal tract containing the microsphere.

The adaption ratio (simulated/measured mean dose) was almost perfect for individual biopsies, on average 0.99 ± 0.025. When comparing the mean absorbed dose distribution of the sub-sample of 16 biopsies (measured activity) with the 41 biopsies (measured activity), it is obvious that the former is not a representative sample, regarding macroscopic distribution (mean: 38 ± 21 vs. 47 ± 34 Gy, median 36 vs. 38 Gy), thus with an overall adaption ratio (sub-sample/sample mean absorbed dose) of only 0.81. By weighting the macroscopic influence of the 16 biopsies, the adaption of the mean is better (mean: 43 ± 38 vs. 47 ± 34 Gy, median: 36 vs. 38 Gy), with an overall adaption ratio (weighted-sub-sample/sample mean absorbed dose) of 0.91, but it is still far from perfect. The reason for this is that the regions with the highest mean absorbed dose were not included in the microscopic sample, as cluster tracking through sequential sections was complicated for biopsies with a high concentration of microspheres. Therefore, the biopsy with the highest mean absorbed dose (i.e., 84 Gy) was used to represent the macroscopic dose distribution >67 Gy. Including biopsies with mean absorbed doses >84 Gy would most certainly extend the “tail” in the high absorbed dose spectrum and slightly increase the proportion of absorbed doses >100 Gy. However, this would not have a large impact on the distribution in the lower part of the spectrum, which is the most critical for the risk of adverse effects and overall survival of the liver following SIRT. We expect that even larger clusters would contain a major proportion of the activity in biopsies with a higher mean absorbed dose and, therefore, an even stronger absorbed dose gradient in such sub-regions.

Our analysis of microsphere distribution was performed in liver tissue resected only 9 days after injection, and we did not note any morphological changes due to irradiation. Earlier studies on human liver tissue were performed months after injection, which probably allowed morphological changes and delocalisation of the microspheres [24,30]. To the best of our knowledge, similar studies of the detailed microsphere distribution soon after injection time have not been published. We performed three similar surgical procedures as described in this work. The autoradiography and biopsy activity measurements in these patients revealed similar macroscopic distributions and were in agreement with previously published results [22,24,30]. Furthermore, the clustering tendency of microspheres in arterioles and small arteries were similar between the patients (data not shown), but due to the limited amount of tissue available for two of these patients, we were only able to perform the extended light microscopic analysis for one patient. Accordingly, it would be beneficial to validate our data in liver tissue resected soon after injection, before morphological changes occur.

Cremonesi et al. [1] previously pointed out challenges in the field of radioembolisation treatments that are relevant for both resin and glass spheres. Dosimetry on a microscopic level is crucial for creating reliable radiobiological models capable of explaining and predicting radiobiological effects and risks. Some authors have described microscopic distributions by investigating explanted liver tissue [25,31] or using simulations [26,43]. Extensive cluster lodgings in larger arterioles and smaller arteries may challenge the previous hypothesis of a rather uniform microscopic microsphere distribution throughout the liver parenchyma for resin spheres [8,10,26,44,45], as the tendency of larger clusters to contain a major proportion of the total activity seems to increase exponentially with mean absorbed dose. As the resin spheres and glass spheres are similar in size, the clustering tendency should be the same for 120 Gy regions with glass spheres and 2.4 Gy regions with resin spheres (the same concentration of spheres given a 50-times higher specific activity for glass spheres). A microsphere concentration causing an absorbed dose level that low is rare for resin spheres and was not investigated in our samples.

Extrapolation of the relationship between the mean cluster size and absorbed dose (*c.f.* Figure 5) of 2.4 Gy gives a mean cluster size of 5.7 and a maximal cluster size of 12 microspheres per cluster. This extrapolation of clinical data resembles the simulation results reported by Walrand et al. [26] (mean cluster sizes with 4 - 5 spheres per cluster, and maximal cluster size of 10 microspheres per cluster). However, the assumption that all microspheres will be located at the terminal portal artery will predict a more uniform absorbed dose distribution in the liver parenchyma for an increased number of microspheres, which contradicts our observed clinical results. We found that an increased number of injected spheres will cause increasing non-uniformity due to microsphere trapping in larger arterioles or small arteries, hampering microsphere transport to the terminal portal artery. Therefore, future extension of the novel model proposed by Walrand et al. [26] should include the probability of upfront microsphere trapping in the arterial tree. Such model extension would be beneficial for increased precision in the explanation and prediction of radiobiological effects and optimisation of radioembolisation treatments.

The macroscopic and microscopic non-uniformity of the absorbed dose distributions may be valuable information for explaining the tolerance of a relatively high mean absorbed dose in the entire liver parenchyma. Strong dose gradients within individual biopsies will result in a systematic non-uniformity and, thus, a lower mean absorbed dose to the parenchyma regions that are not very close to large clusters. Such systematic inhomogeneity in the absorbed dose may be beneficial for the radiation tolerance of the parenchyma. A higher concentration of spheres needed for treatment with lower activity per sphere, i.e., resin spheres, increases the absorbed dose non-uniformity within the liver parenchyma, competing with the increase in non-uniformity due to the higher activity per microsphere of glass spheres. Therefore, absorbed dose non-uniformity is not just a question of activity per microsphere, but also of the concentration of microspheres.

The linear regression of the increasing CV of absorbed dose with mean absorbed dose was weak (R^2^ = 0.34). The regression was however significant (*p* < 0.05) and the only significant (*p* > 0.05) outlier was found within the dependent variable data set (for CV 0.87); no significant outlier (*p* > 0.05) was found within the independent variable data set. Excluding the data point with the significant outlier (59 Gy; 0.87), would improve the regression (R^2^ = 0.37, *p* < 0.05). Given the high probability that the largest clusters might be underestimated, since only 15 sequential sections were investigated, it is probable that the actual dependence is stronger. Future investigations should use samples with more than 15 subsequent 30 μm sections, which most probably will result in improved correlation between the CV and mean absorbed biopsy doses.

The gathered data in this study are strong enough, however, to show that cluster distributions are homogenised by cross-fire [46] to only a limited extent. The resulting absorbed dose distributions are non-uniform (high CVs) throughout all 16 simulated sub-volumes (Figure 7) and there is a tendency of an increase in relative small-scale absorbed dose non-uniformity with increasing biopsy mean absorbed dose.

## Conclusion

In conclusion, the strong absorbed dose heterogeneity verified in this study was due to varying degree of sphere aggregation. The sphere clusters were located in both arterioles and small arteries and increased in size with increasing number of spheres per mass unit. The absorbed dose simulations showed that also the absorbed dose inhomogeneity increased with increasing absorbed dose. These results contradict earlier modelling studies and may have important influence in explaining the radiobiological situation in RE therapy.

### Ethical approval

All procedures performed in studies involving human participants were approved by the Regional Ethical Review Board in Gothenburg, Sweden, and were in accordance with the 1964 Helsinki declaration and its later amendments or comparable ethical standards.
